# Editorial: Highly efficient bioconversion of biomass waste: From theory to industry

**DOI:** 10.3389/fbioe.2023.1147993

**Published:** 2023-04-11

**Authors:** Qingyang Lyu, Liyan Song, Yen Wah Tong, Wenguo Wang, Jun Zhou, Zhiying Yan

**Affiliations:** ^1^ Chengdu Institute of Biology, Chinese Academy of Sciences (CAS), Chengdu, China; ^2^ School of Resources and Environmental Engineering, Anhui University, Hefei, China; ^3^ Department of Chemical and Biomolecular Engineering, National University of Singapore, Singapore, Singapore; ^4^ Biogas Institute of Ministry of Agriculture and Rural Affairs, Chengdu, China; ^5^ College of Biotechnology and Pharmaceutical Engineering, Nanjing Tech University, Nanjing, China

**Keywords:** biomass waste, poly 3-hydroxybutyrate, humic substances, sugar recovery, biochar

Biomass waste, including stalks, manures, garden trash, food residues, and sewage sludge, have become an emerging environmental Research Topic worldwide. However, there are other options and biomass waste could be used to produce energy, fuels, fertilizers, and food, as well as valuable chemicals through appropriate bioconversion. With the advantages of having a low cost and there being various application scenarios, the bioconversion of biomass waste into valuable products is a sustainable and environmentally-friendly manufacturing process compared with traditional petroleum-derived production. In recognition of the increasing amount of literature being published on biomass bioconversion, this Research Topic of Frontiers in Bioengineering and Biotechnology collects diverse papers associated with the efficient utilization of biomass waste, including poly 3-hydroxybutyrate fermentation, humic bioconversion, sugar recovery, and the application of biochar. There are two reviews in this Research Topic. The first review explores poly 3-hydroxybutyrate (PHB) fermentation from biomass waste, and the second demonstrates humic substances during aerobic bioconversion. In their contribution, Zhang et al. summarize the latest PHB production techniques *via C. necator* fermentation. PHB is an important biopolymer that is used to produce bioplastics for the substitution of petroleum-derived plastic materials. Here, the metabolism pathway of PHB biosynthesis is illustrated, and the economic effectiveness of different PHB fermentation feedstocks are compared. After discussion of the current enhancing strategies during *Cupriavidus necator* fermentation, the authors propose guidelines for PHB yield and improving productivity in the future.

Hu et al. review the bioconversion from biomass waste to humic substances (HSs) *via* aerobic composting (AC) and hydrothermal treatment (HT). HSs were commonly used in soil remediation, which is generated at a very low rate in nature. Thus, AC and HT were employed as artificial humification strategies to accelerate this process. The author summarizes the diverse condition parameters which affect AC and HT, including temperature, feedstock type, pH value, and pretreatment method. The corresponding mechanism of HSs formation during AC and HT are also proposed. Considering the advantage of AC and HT, HT could be used as a pretreatment method before AC to expedite the humification process.

Biomass waste is a potential reservoir of energy and substance resources. The study by Cheng et al. focuses on the higher sugar recovery from *M. sinensis*, a lignocellulosic grass in China, with lower chemical reagent cost. To get a better delignification and saccharification effect, several surfactants were added during alkaline pretreatment, and the results indicated that PEG 2000 (poly ethylene glycol) at 1% content was suitable for sugar recovery from *Miscanthus sinensis*. In enzymatic hydrolysis, Tween 80 was the best surfactant. The addition of 1% Tween 80 could slightly improve the sugar yield at a lower enzyme loading, which could be an effective way to save costs during bioethanol industrial production.

In the treatment with another lignocellulosic waste sugarcane bagasse (SCB), Wang et al. explore the possibility of soluble sugar extraction and adsorbent utilization. Interestingly, soluble sugars were extracted from SCB with simple solid-liquid separation, and the concentration reached as high as 0.4 g/g SCB. After soluble sugar extraction, the debris could be further enzymatically hydrolyzed to generate glucose from cellulose, and the addition of persulfate could enhance this process. Meanwhile, both SCB and SCBE (by-products after SCB sugar extraction) were shown to be capable of adsorbing methylene blue and Cu^2+^, indicating applications for dye and metal ion elimination from wastewater.

Three studies in this Research Topic present research results about biochar, a multifunction substrate derived from diverse biomass waste. Zhang et al. report on a low-cost adsorbent for cationic dyes, which is a kind of calcium-rich biochar pyrolyzed from spent mushroom substrate. Higher pyrolysis temperature endowed the biochar with higher Ca content and larger specific surface area, resulting in strengthened adsorption capacities for both Malachite Green oxalate and Safranine dyes. This direct pyrolysis of spent mushroom substrate without extra modification indicated a promising way for dyeing wastewater treatment.

Qin et al. employed hydrophobic biochar, which was used in the amendment of landfill cover soil. The water retention capacity of biochar was weakened by a modifier to generate hydrophobic biochar. After mixing the hydrophobic biochar and landfill cover soil, the CH_4_ was totally removed with CH_4_ content that varied from 5% to 25%. Compared with the common biochar, the addition of hydrophobic biochar reduced the moisture of the soil, which subsequently enhanced the abundance of methane-oxidizing bacteria (MOB), resulting in a higher methane removal capacity, especially in the upper and lower layer of soil.

Composting is a common method of bioconversion of diverse biomass waste substrates, especially agricultural waste, such as livestock manure, and straw. However, the antibiotics and antibiotic resistance genes (ARGs) dwelling in the manure possesses great health risks to human beings. The elimination of these emerging pollutants is a research hotspot and has been the subject of numerous studies. The study by Tong et al. demonstrates the application of biochar during the co-composting of pig manure and corn straw, in which the addition of biochar improved the pile temperature and prolonged the high temperature period, leading to the enhanced removal efficiency of antibiotics, as well as a reduction of ARGs and MGEs. This could be an option for antibiotic and ARGs management in other composting systems.

Although the research studies in this Research Topic offer scientific guidance for the bioconversion of biomass waste, they are not comprehensive and exhaustive. Currently, the conversion and utilization of biomass waste still result in extensive secondary pollution, incomplete conversions, and low-value products, for example, approaches such as aerobic composting and anaerobic digestion. With the rapid development of engineering and synthetic biology, the bioconversion of biomass waste should be upgraded and steerable. A new reactor with precise controllable parameters, and artificially synthetic strains or microflora with high efficiency and broad-spectrum feedstock should be explored and employed in the future, as should simple and economic production routes for high-value compounds. Overall, we are very grateful to the authors who have contributed to this Research Topic for sharing their significant works and ideas. We would also like to thank the reviewers for their critical and professional reviews. We hope the research collected here will provide novel, inspired, and useful traits on the bioconversion of biomass waste, and lay the foundations for potential industrial applications.

